# Jumping Dynamics of Cyanomethyl Radicals on Corrugated Graphene/Ru(0001) Substrates

**DOI:** 10.1021/acs.jpcc.4c06312

**Published:** 2024-12-05

**Authors:** Michele Pisarra, Juan Jesús Navarro, Cristina Díaz, Fabian Calleja, Amadeo L. Vázquez de Parga, Fernando Martín

**Affiliations:** † Instituto IMDEA Nanociencia, Calle Faraday 9, 28049 Madrid, Spain; ‡ Departamento de Química, Módulo 13, Universidad Autónoma de Madrid, 28049 Madrid, Spain; § Dipartimento di Fisica, Università della Calabria and INFN-gruppo Collegato di Cosenza, Via P. Bucci, Cubo 30 C, 87036 Rende, Italy; ∥ Departamento de Física de La Materia Condensada, Universidad Autónoma de Madrid, 28049 Madrid, Spain; ⊥ Departamento de Química Física, Facultad de CC. Químicas, Universidad Complutense de Madrid, 28040 Madrid, Spain; # Condensed Matter Physics Center (IFIMAC), Cantoblanco, 28049 Madrid, Spain

## Abstract

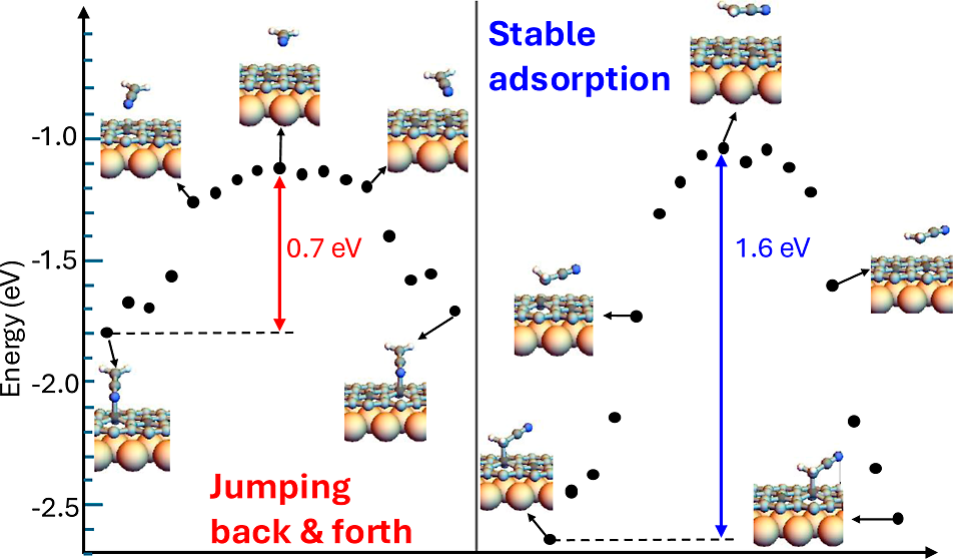

Graphene adsorbed on Ru(0001) has been widely used as a template for adsorbing and isolating molecules, assembling organic-molecule structures with desired geometric and electronic properties and even inducing chemical reactions that are challenging to achieve in the gas phase. To fully exploit the potential of this substrate, for example, by being able to tune a graphene-based catalyst to perform optimally under specific conditions, it is crucial to understand the factors and mechanisms governing the molecule–substrate interaction. To contribute to this effort, we have conducted a combined experimental and theoretical study of the adsorption of cyanomethyl radicals (−CH_2_CN) on this substrate below room temperature by performing scanning tunneling microscopy experiments and density functional theory simulations. The main result is the observation that some −CH_2_CN molecules can jump back and forth between adsorption sites, while such dynamics is not seen above room temperature. We interpret this finding as the consequence of the molecules being adsorbed on a secondary adsorption configuration in which they are bound to the surface through the nitrogen atom. This secondary configuration is much less stable than the primary one, in which the molecule is bound through the −CH_2_ carbon atom due to an sp^2^-to-sp^3^ hybridization transition. The secondary configuration adsorption is achieved only when the cyanomethyl radical is deposited at low temperature. Increasing the substrate temperature provides the molecule with enough energy to reach the most stable adsorption configuration, thereby preventing the jumping.

## Introduction

Graphene grown on transition metal substrates is considered a versatile template for adsorbing molecules and molecular complexes,^1^ allowing for the study of their properties and the formation of new structures with unique characteristics. The adsorption of graphene has been achieved on numerous transition metals, including Ni(111),^2^ Ir(111),^3−6^ Cu(111),^7^ Pt(111),^8,9^ Rh(111),^10^ Re(0001),^11^ Co(0001),^12,13^ and Ru(0001).^14−19^ Graphene adsorbed on Ru(0001), hereafter termed Gr/Ru, is by far the most studied one. In this system, the lattice constant mismatch between graphene and Ru(0001) causes the appearance of a moiré pattern, resulting in a modulation of the C–Ru interaction across the unit cell. In the higher regions of the moiré pattern, the graphene is physisorbed, whereas in the lower regions, it is chemisorbed. This modulation of the C–Ru chemical binding induces significant vertical corrugation, both structural and electronic, of the graphene layer. This affects the surface dipole and surface potential^20^ and also modulates the graphene local density of states near the Fermi level resulting in overall n-doping of the graphene layer.^15^

These unique characteristics of the Gr/Ru system make it an appealing substrate for the development of new molecular physics and chemistry. For example, graphene can act as a buffer layer, decoupling the molecule electronic states from those of the underlying ruthenium, enabling the recording of high-resolution images of the molecular orbital of numerous molecules, from pentacene and fullerene^21^ to molecules with richer chemical structure and composition, such as perylene-3,4,9,10-tetracarboxylic-3,4,9,10-dianhydride (PTCDA),^21^ 7,7,8,8-tetracyano-*p*-quinodimethane (TCNQ), and tetrafluoro-tetracyano-*p*-quinodimethane (F4-TCNQ).^22^ Experiments and theoretical calculations have shown that upon adsorption of acceptor molecules like TCNQ and F4-TCNQ on Gr/Ru, a whole electron can be transferred from the n-doped sites of the substrate to the molecule, while the latter maintains a flat geometry.^22,23^ TCNQ can also use Gr/Ru as a template to build two-dimensional (2D) structures, which form as a result of the competition between the intermolecular and molecule–surface interactions. In particular, this acceptor molecule takes advantage of the n-doped nature of graphene to form 2D arrays with long-range magnetic order.^23,24^ 2D structures have also been achieved with other molecules. For example, PTCDA molecules form a 2D herringbone pattern^25^ as a result of the strong intermolecular interactions associated with C–H···O-type hydrogen bonds. Molecules such as 2,4′-bis(terpyridine) (2,4′-BTP) and 2-phenyl-4,6-bis(6-(pyridin-3-yl)-4-((pyridin-3-yl)pyridin-2-yl)pyrimidine) (3,3′-BTP) preferentially occupy the low regions of the moiré forming linear or ring-like structures.^25,26^ Phthalocyanines (*Pc*), such as FePc, NiPc, and H_2_Pc, can also form 2D structures, such as Kagome lattices that follow the periodicity of the Gr/Ru moiré pattern.^27−29^ These structures are ideal models for studying spin frustration related to spintronic applications. Also interesting for molecular spintronic could be the 2D structures formed by endohedral fullerenes with magnetic spins.^30^ They form hexagonal supramolecular structures similar to those formed by simple fullerenes.^31^

Beyond its applications as a template, in recent years, Gr/Ru has also been proposed as an efficient catalyst for promoting chemical reactions; for example, its potential to promote the synthesis of long-chain acenes via on-surface photogeneration from α-bis-diketone precursors.^32^ The striking efficiency of this process has been rationalized in terms of the appearance of a new unoccupied electronic state above the Fermi energy, resulting from the interaction between the graphene image state and the ruthenium surface resonance.^33^ Gr/Ru has also been shown to efficiently promote chemical reactions involving reversible C–C bond formation between cyanomethyl radicals (−CH_2_CN) and TCNQ molecules.^34^ Very recent experimental work showed that sulfur compounds such as sulfur dioxide (SO_2_)^35^ and thiophene (C_4_H_4_S)^36^ adsorb and dissociate efficiently on Gr/Ru, the latter promoting the formation of gaseous acetylene (C_2_H_2_) and thioketene (C_2_H_2_S).

Surface temperature can significantly impact the molecule adsorption on Gr/Ru. For instance, controlling the surface temperature can induce long-range self-assembled distinctive 2D structures, known as loose-packed and close-packed formations, of pentacene on Gr/Ru.^37^ Moreover, temperature is the crucial parameter for achieving site-selective patterning of the Gr/Ru substrate with −CH_2_CN radicals.^38,39^ These studies have shown that at room temperature or above, −CH_2_CN radicals covalently bind to the graphene through the carbon bonded to the H atoms due to an sp^2^-to-sp^3^ hybridization transition. However, if the substrate temperature is kept below room temperature during the molecule deposition, scanning tunneling microscopy (STM) measurements reveal that some −CH_2_CN molecules jump back and forth between adsorption sites, which may seem counterintuitive, as one would expect that the mobility of the adsorbed molecules should be favored by increasing the temperature and not the other way around.

Our goal here is to elucidate the physical mechanisms behind the behavior observed for cyanomethyl radicals adsorbed on Gr/Ru at a low deposition temperature. To this aim, we have conducted a combined study using STM measurements and density functional theory (DFT) simulations. Our analysis reveals that the behavior of −CH_2_CN at low deposition temperature is related to the accessibility of a somewhat unstable bonding configuration, in which the nitrogen atom binds to the graphene with minimal change in the molecule’s geometry. This configuration is unattainable at room temperature.

## Methods

The experiments have been carried out in an ultrahigh vacuum (UHV) chamber with a base pressure of 5 × 10^–11^ Torr equipped with a low-temperature STM and facilities for sample and tip preparation. The graphene layer was prepared by chemical vapor deposition using an ethylene partial pressure of 8 × 10^–8^ Torr at a sample temperature of 1150 K. Exposure to cyanomethyl radicals was achieved by introducing acetonitrile molecules in the UHV chamber via a leak valve at a partial pressure of 1 × 10^–6^ Torr, using a Bayard–Alpert pressure gauge calibrated for N_2_ as a source for the radicals.^38,39^ All STM measurements were performed using liquid N_2_ in both jackets of the cryostat, attaining a base temperature of 80 K. The low-temperature exposure to cyanomethyl was conducted by transferring the sample from the cold STM to the main manipulator, at room temperature, and starting the exposure as fast as possible (i.e., within a few minutes). Hence, we estimate an actual sample exposure temperature between 100 and 150 K. The threshold analysis of the STM video frames has been done with the ImageTank software.^40^

The simulations have been carried out using DFT within the projector augmented wave approach,^41^ as implemented in the VASP code,^42−44^ using the Perdew–Burke–Ernzerhof (PBE) exchange correlation functional^45^ and the Tkatchenko–Scheffler^46^ corrections, to account for weak dispersion forces. We adopted a 400 eV plane wave cutoff and a total energy threshold of 10^–5^ eV for the self-consistent field calculations, and taking into account the size of the unit cell, we have limited the reciprocal space sampling to the Γ point. The Gr/Ru system was modeled by a three-layer-thick (10 × 10) Ru(0001) slab with a (11 × 11) graphene adsorbed on one side, in line with the existing literature.^47−50^ The geometry optimization has been carried out relaxing the coordinates of the cyanomethyl radical, all C atoms of graphene, and the Ru atoms of the topmost Ru layer, until the maximum force on the active atoms was less than 0.01 eV/Å. The transition-state calculations have been carried out using the nudged elastic band (NEB) method,^51,52^ as implemented in VASP. A total of 15 images have been used, adopting a force threshold of 0.05 eV/Å. For these more demanding calculations, the active degrees of freedom have been limited to only the molecule atoms and the 2 graphene atoms involved in the molecule–substrate bond.

## Results and Discussion

**Figure 1:**
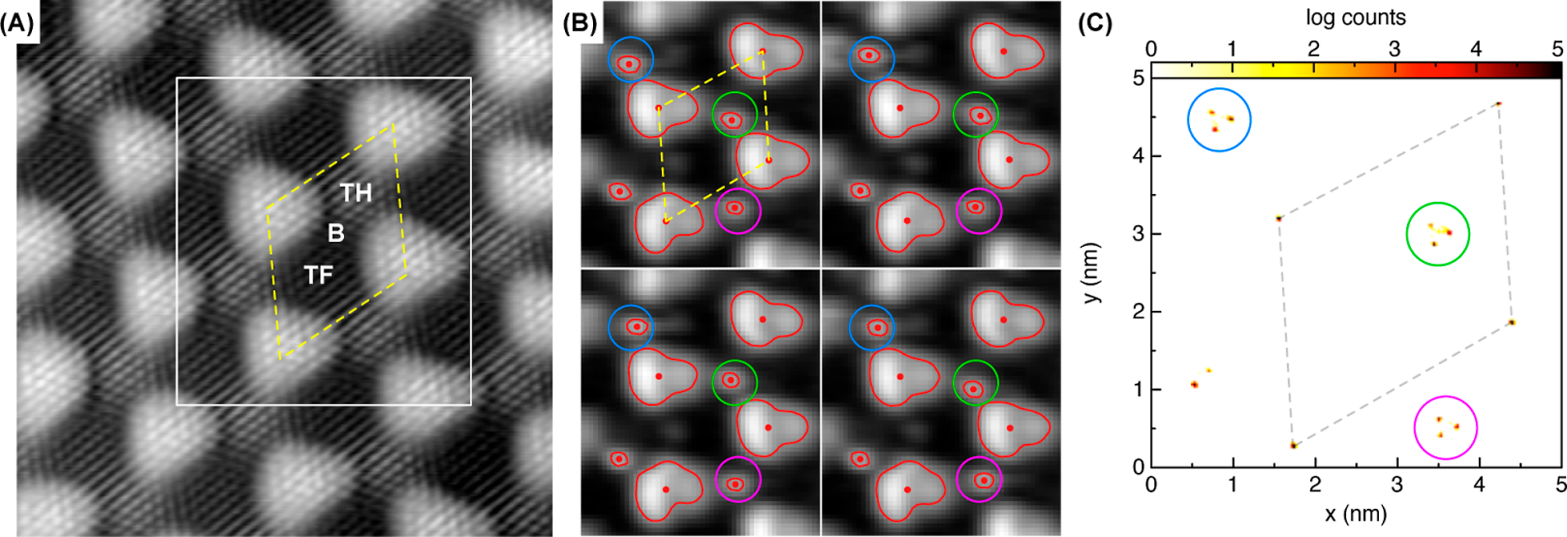
(A) STM image of a pristine Gr/Ru surface (10 × 10 nm^2^, acquired at bias voltage *V*_b_ = 10 mV and tunneling current *I*_t_ = 700 pA); the yellow dashed line highlights the moiré unit cell, and the three inequivalent regions of the low area of the moiré are also identified as Top-Hcp (TH), Bridge (B), and Top-Fcc (TF). (B) Four frames taken from the video given as the Supporting Information (5 × 5 nm^2^, acquired at *V*_b_ = −1 V and *I*_t_ = 30 pA current). The cyanomethyl radicals appear as bright bumps in the TH regions,^38^ three of which have been highlighted in blue, green, and magenta, respectively. Constant-height contours obtained from each frame are represented as red lines, with their corresponding centers of mass marked as a red dot. The constant-height threshold has been carefully adjusted to map both moiré hills and the radicals. Tiny lateral displacements of the radicals can be observed comparing the different frames. (C) 2D histogram of all centers of mass as a function of the lateral position, obtained from the 506 frames of the complete video. The total number of events, indicative of the relative permanence time, is represented in a logarithmic color scale. The three TH positions highlighted in panel B are encircled in green, blue, and magenta, respectively, following the same color code. The moiré unit cell is represented as a gray dashed line.

An atomic resolution STM image of the pristine Gr/Ru moiré pattern is shown in Figure 1A, where we observe the physisorbed zones of the moiré emerging as bright protuberances organized in a hexagonal lattice. In the chemisorbed region (darker area), it is possible to distinguish three different zones, due to the different electronic corrugation,^49,53^ namely, (i) the Top-Hcp (TH) region, in which one of the two C atoms characterizing the graphene lattice sits on top of a Ru atom and the other is adsorbed on the *hcp* hollow site of the surface; (ii) the Top-Fcc (TF) region, in which one of the two C atoms sits on top of a Ru atom and the other one is adsorbed on the *fcc* hollow site of the surface; and (iii) the Bridge (B) region, in which the C–C bond is on top of the Ru atoms. As already discussed in the literature,^38,39^ on this substrate, the highly reactive cyanomethyl radicals, created from acetonitrile (CH_3_CN) using an ion gauge, covalently bind with the carbon atoms of the graphene layer. A precise control in the adsorption site can be achieved if the substrate is kept at a surface temperature of 375 K (or higher) during the cyanomethyl deposition, with more than 98% of the molecules adsorbed on the TH regions of the moiré.^39^ However, lowering the substrate temperature during the deposition leads to progressively less site selectivity and more disorder. In fact, a very interesting phenomenon is observed when the cyanomethyl deposition is carried out, keeping the substrate below room temperature. Beyond the disorder, some cyanomethyl radicals appear to be unstable in their position when imaged by the STM. In the video provided as the Supporting Information, it is possible to see the cyanomethyl radicals bouncing from site to site in the successive 506 total frames, four of which are presented in Figure 1B. After correcting the thermal drift, a threshold analysis has been performed on every frame of the video, identifying the constant height contours related to both physisorbed moiré hills (used as a reference for drift correction) as well as the unstable cyanomethyl radicals present in the scanning area. These contours are represented in Figure 1B (and in the video) as red lines with their corresponding centers of mass represented as red dots. Three of the available TH sites are highlighted in blue, green, and magenta, respectively. In Figure 1C, we provide a 2D histogram of the centers of mass of all of the contours resulting from the 506 frames of the video, represented in a logarithmic color scale. The previously highlighted TH sites are encircled again in blue, green, and magenta, and the moiré unit cell is represented as the gray dashed line. Within the different TH sites, the cyanomethyl radical can occupy three different distinct positions. Interestingly, these three positions form an equilateral triangular shape with a measured side of 230 ± 20 pm, in agreement with the lattice parameter of graphene of 246 pm. A further inspection of the video reveals no significant meaningful jump pattern; the radicals change position randomly due to the interaction with the STM tip, which can transfer energy to the molecules during its scanning motion. At this point, it is important to emphasize that the bouncing molecules were not observed in depositions conducted at surface temperatures above 300 K.

**Figure 2:**
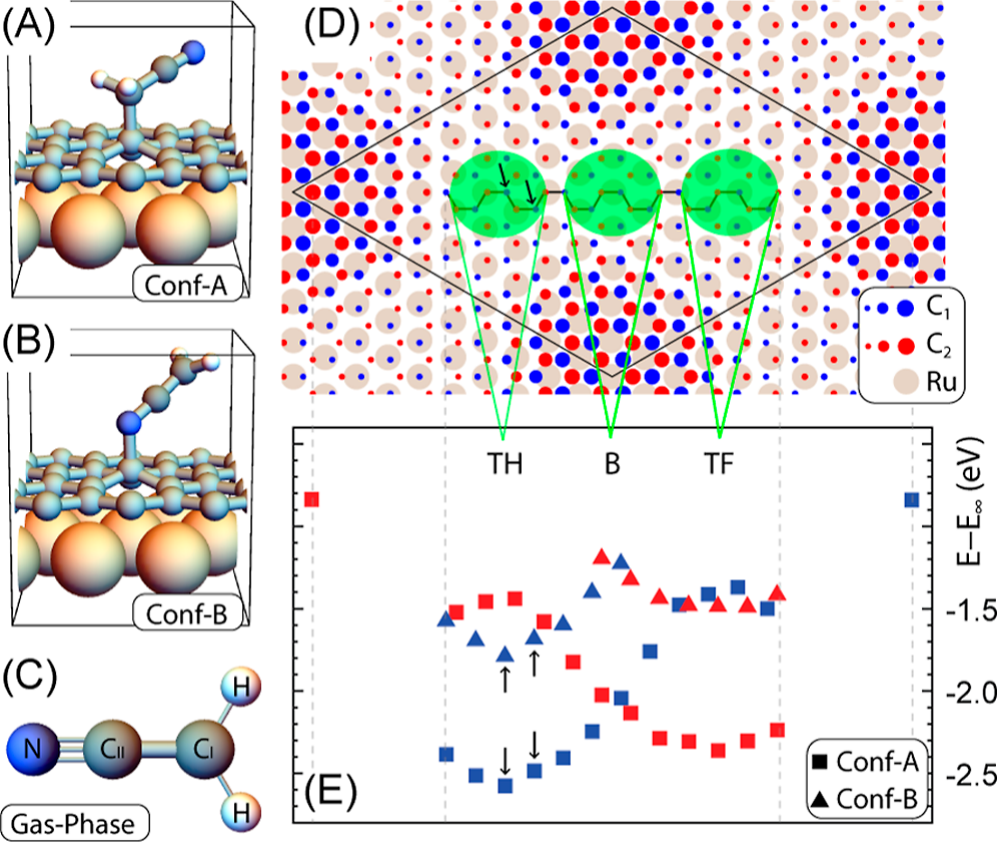
(A,B) Geometry configuration in which the cyanomethyl radical is adsorbed by means of a C–C bond (Conf-A) or a N–C bond (Conf-B). (C) – CH_2_CN^•^ in the gas phase. (D) Top view of the G(11 × 11)/Ru(10 × 10) moiré pattern (only the topmost Ru layer is shown): the two carbon sublattices of graphene are distinguished; the size of the points depicting the C atoms is proportional to the distance from the Ru layer; the inequivalent zones in the low part of the moiré are highlighted; and the armchair lines connect the C atoms used in the adsorption study. The arrows identify the adsorption sites used in NEB calculations. (E) Total energy of the adsorbed cyanomethyl molecule on Gr/Ru: the color of the point reflects the carbon sublattice as in panel (D); the zero of the energy scale is set using as reference a configuration in which the cyanomethyl radical is put far away from an unperturbed Gr/Ru surface with total energy equal to *E*_∞_. The arrows mark the energies of the extrema (images 0 and 16) of the NEB calculations (cf. Figure 3).

To gain deeper insight into the adsorption mechanism of the cyanomethyl radical on Gr–Ru and the intriguing phenomenon of the bouncing molecules, we conducted a comprehensive DFT-based adsorption study. As previously shown in ref (38), two possible configurations exist in which the cyanomethyl radical forms a covalent bond with Gr/Ru (see Figure 2A,B). Before analyzing these configurations in detail, it is important to note that in the gas phase (cf. Figure 2C), the cyanomethyl radical is perfectly planar. The central carbon atom (C_I_) exhibits sp^2^ hybridization and, due to the unpaired electron, forms a shortened single bond with the carbon atom, C_II_, which in turn forms a triple bond with the nitrogen atom. When the cyanomethyl radical is adsorbed on Gr/Ru in the hereafter called Conf-A (shown in Figure 2A), it forms a covalent bond with the surface through the C_I_ atom. Notably, in this configuration, the molecule is no longer planar and the C_I_ atom exhibits sp^3^ hybridization. In the hereafter called Conf-B (shown in Figure 2B), the cyanomethyl radical also forms a covalent bond, this time through the N atom. In this configuration, the molecule retains a flat geometry with the primary modification being a slight elongation of the C_II_–N bond. Both configurations A and B exhibit positive binding energies, with Conf-A being approximately 1 eV energetically more favorable.^38^

The binding energy of the cyanomethyl radical is strongly influenced by the adsorption site on the Gr/Ru surface. This can be clearly seen in Figure 2D,E, where we present the total energy of the covalently bonded cyanomethyl radical (either Conf-A or Conf-B) at various sites on the Gr–Ru surface. Notably, the approximate 1 eV difference in binding energy between Conf-A and Conf-B is consistently observed across all of the investigated adsorption sites. More importantly, regardless of the adsorption configuration, the TH region consistently exhibits the lowest energies, aligning well with the experimental results. Additionally, we observed a significant difference in energy between the two inequivalent sublattices of graphene. The lowest energy is always attained when the graphene carbon atoms involved in the covalent bond are located in a hollow site of the Ru surface. A closer examination of Figure 2D,E reveals that in the TH region, the lowest energies correspond to the carbon atoms of sublattice 1 (C_1_, blue markers) situated in the hollow sites of the Ru surface. In the TF region, the C_2_ atoms are located in the hollow sites of the surface, resulting in the lowest total energies (red markers). This phenomenon can be understood by noting that the graphene carbon atom participating in the covalent bond with the cyanomethyl radical is slightly elevated compared to its neighboring atoms (cf., Figure 2A,B). This elevation implies an energy cost, which is reflected in the final total energy. The carbon atoms situated directly above the Ru atoms are more strongly bonded to the surface, requiring higher energy to lift them compared to the more loosely bonded hollow carbon atoms. Interestingly, in the Bridge region, where the atoms from the two sublattices are equidistant from the underlying Ru atoms, no significant difference is observed in the absorption energy of the cyanomethyl radical between the two sublattices.

Figure 2E also shows that adjacent adsorption sites within the same sublattice in the Top-Hcp region (in either Conf-A or Conf-B) result in minimal energy differences. Thus, we can conclude that the observed back and forth bouncing occurs between adsorption sites of the same sublattice, specifically involving binding of the molecule to a carbon atom located in an *hcp* hollow site of the Ru surface. This scenario is consistent with the previously mentioned experimentally measured jump distances, of 230 ± 20 pm.

**Figure 3:**
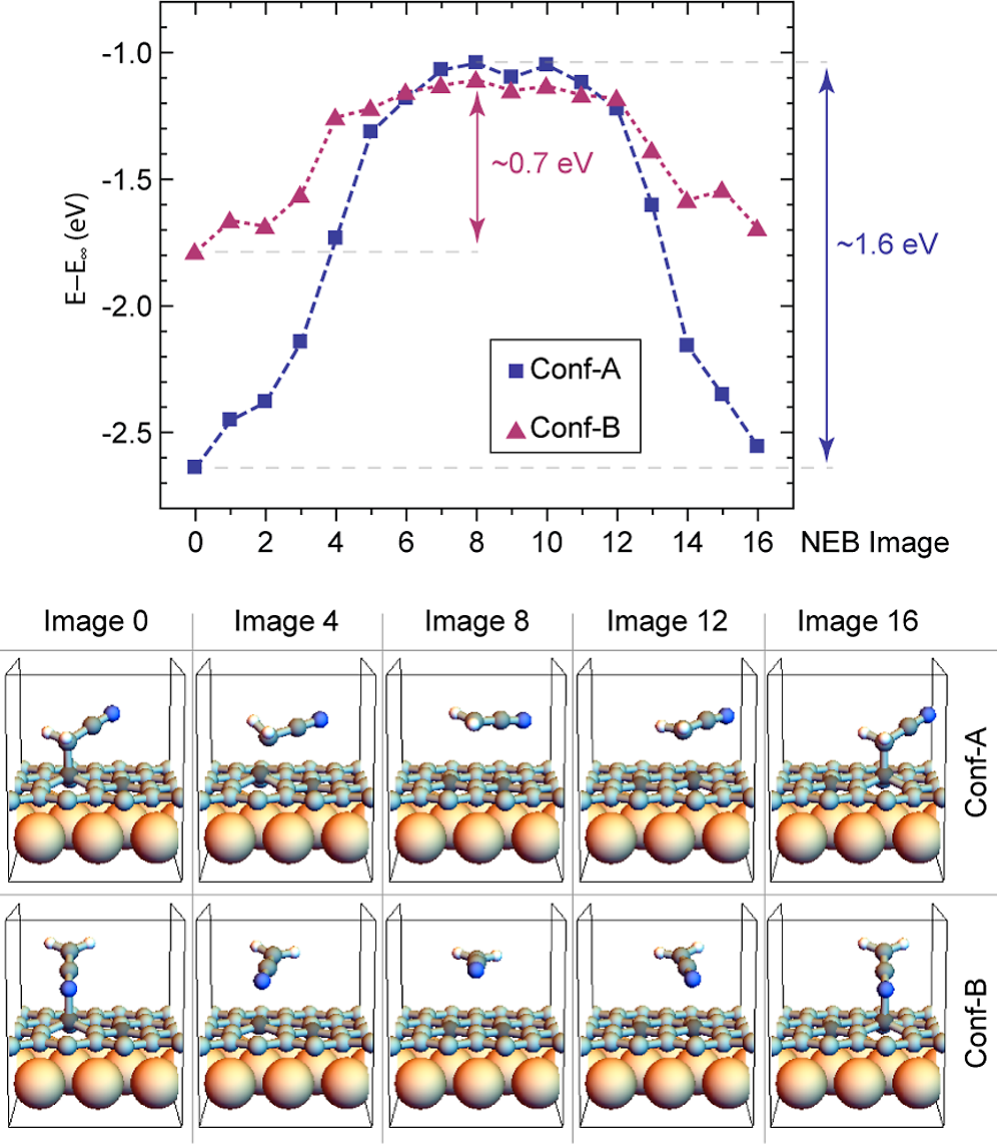
NEB calculation between two adjacent adsorption sites on Gr/Ru for the cyanomethyl radical in Conf-A and Conf-B.

To gain deeper insight into the stability of each adsorption configuration, we conducted a NEB study of the transition state between two adjacent sites in the same carbon sublattice, either in Conf-A or Conf-B. As shown in Figure 3, a barrier of approximately 1.6 eV must be overcome to shift the cyanomethyl radical from the most stable adsorption site to the adjacent site within the same sublattice when the molecule is adsorbed in Conf-A. On the other hand, for Conf-B, a significantly lower barrier of approximately 0.7 eV is observed. Thus, the results shown in Figure 3 indicate that bouncing of the molecules occurs when the cyanomethyl radical is adsorbed in Conf-B.

A final phenomenon that requires explanation is why the bouncing molecules were observed only during deposition at low substrate temperatures. As discussed above, the formation of Conf-A involves a structural change in the cyanomethyl radical, with the C_I_ atom shifting its hybridization from sp^2^ to sp^3^. A vibrational analysis of the gas-phase molecule shows that the out-of-plane wagging of the C_I_–H_2_ group, associated with the sp^2^-to-sp^3^ transformation of the C_I_ atom, has an energy of approximately 0.089 eV. Conversely, Conf-B is associated with a slight deformation of the C_II_–N bond. The in-plane and out-of-plane bond bending vibrations related to this deformation have energies of approximately 0.046 and 0.052 eV, respectively. Therefore, forming Conf-A requires initiating a vibration in the molecule with roughly twice the energy needed to achieve Conf-B. When the substrate temperature is sufficiently high, the substrate can transfer a significant amount of energy to the incoming cyanomethyl radical, allowing it to explore a wider phase space until reaching the global minimum represented by the adsorption in Conf-A, where it is highly stable. Conversely, when the substrate temperature is low, not all cyanomethyl molecules reach stable Conf-A; some remain in less favorable Conf-B. However, in Conf-B, the low energy barrier allows the molecule to jump from one site to an adjacent one when interacting with the STM tip.

## Conclusions

We studied the adsorption of cyanomethyl radicals on graphene grown on Ru(0001) using STM. Our results reveal a striking jumping behavior of some of these molecules when deposited below room temperature, with the jumping always occurring within the same substrate sublattice. To rationalize these experimental results, we performed DFT-based simulations. Our theoretical results indicate that only some molecules jump because, from the two possible adsorption configurations, only that in which the molecule is bound to the substrate through the N atom (Conf-B) can do it. The reason is that it does not require a change in the molecule C_I_’s hybridization. The jumping of molecules adsorbed through the C_I_ atom (Conf-A) requires overcoming an energy barrier twice as large as that of molecules in Conf-B. The jumping is observed only when the deposition is carried out at low surface temperature because, at these temperatures, only the less stable Conf-B configuration is reachable. At room temperature and above, the surface transfers enough energy to the molecule to promote the sp^2^-to-sp^3^ hybridization change required to reach the most stable Conf-A adsorption, from which the molecules cannot jump. As the only role of the STM tip in this study is to provide enough energy to uncover the metastable molecular configuration, we do not expect these conclusions to depend on the specific form of the tip.

In summary, our study shows that deposition of complex molecules on graphene-based materials at low temperatures provides insight into the kinetics and energetics of metastable adsorption configurations that cannot be observed at higher temperatures. Since these configurations can act as intermediate states in catalytic reactions, our study can be considered as a further step toward the development of graphene-based catalysts of chemical reactions involving complex organic molecules.

## Supporting Information Available

Video showing the jumping of the cyanomethyl radicals back and forth between adsorption sites (MP4)
